# Dually Efficacious Medicine Against Fibrosis and Cancer

**DOI:** 10.3390/medsci7030041

**Published:** 2019-03-04

**Authors:** Daohong Chen

**Affiliations:** Research Institute of Biological Medicine, Yiling Pharmaceutical, Beijing 102600, China; daohong@hotmail.com

**Keywords:** dual efficacy, fibrosis, cancer

## Abstract

Although there is a contemporary consensus of managing a severe disease with multi-targeted approach-based therapeutic combinations, it should not be ignored that certain patho-biological pathways are shared by distinct medical conditions and can be exploited to develop an exceptional type of medication conferring a dual efficacy. This article thus presents a spectrum of emerging molecular targets that substantially contribute to the pathogenesis of both fibrotic and neoplastic disorders, including kinase activities, cytokine cascades, and protein dynamics among others. Moreover, recently approved therapeutic agents in this regard have been sorted out to corroborate the drug’s ability upon targeting each one of these molecular pathways to treat fibrosis and cancer simultaneously. It not only streamlines an overlapping mechanistic profile in the pathogenesis across these two medical conditions, but also inspires clinicians and pharmaceutical innovation to tackle concomitant diseases, such as fibrosis and cancer, with an optimally efficacious medication.

## 1. Introduction

As a common pathological aspect in a broad array of chronic diseases, fibrosis is characterized by diminishment of resident parenchymal cells in body organs and activation of fibroblasts with over-production of the extra-cellular matrix (ECM). Fibrotic disorders can affect numerous body systems, eventually resulting in tissue remodeling and organ failure, which consequently contribute to high mortality in idiopathic pulmonary fibrosis (IPF), liver cirrhosis, and systemic sclerosis (SSc), among others [1,2]. To date, although the etiology of fibrotic diseases remains elusive and the clinical presentations of them may appear quite diverse across various organs, there has been a consensus that chronic injury-stimulated core pathogenesis is linked to certain biological signaling pathways including transforming growth factor-β (TGF-β) and platelet-derived growth factor receptor (PDGFR) [3,4].

Clinically, fibrotic disorders are associated with a higher risk of malignant co-morbidity in a dynamic and reciprocal manner. For instance, patients with IPF have a five-fold risk of developing lung cancer, compared to that of general population [5]. In parallel, up to 80% of hepatocellular carcinoma (HCC) cases arise on the basis of liver cirrhosis following the initial pathological injury such as the virus hepatitis [6]. Besides, SSc patients have been noted as having elevated malignant prevalence rates, including lung cancer, breast cancer and lymphoma. In addition, SSc is linked to an increased incidence of Barrett’s esophagus, a well-known precancerous lesion of esophageal adenocarcinoma [7]. Conversely, from the perspective of cancer biology, activated fibroblasts and the over-expressed matrix bearing metalloproteinases (MMPs) represent important components of the tumor microenvironment, to facilitate the invasion and metastasis of malignant cells [6,8]. Moreover, it has been recognized that fibrosis and cancer share several risk factors, such as chronic inflammatory stimulation which plays a pivotal role in the promotion of fibroblast activation and neoplastic proliferation [8]. Besides, recently emerging molecular evidence reveals the presence of oncogenic gene signatures in fibrotic disease [9,10].

Of note, there is a tendency of decreased mortality from non-malignant pathology-based tissue damage in fibrotic disorders in recent years, due to progress in organ-based treatments including endothelin1 (ET1) antagonists for pulmonary hypertension/fibrosis [11] and angiotensin-converting-enzyme (ACE) inhibitors for cardiac failure/renal fibrosis [12]. As a result, an increased risk of cancer is underscored to be a stronger co-morbidity concern calling for better medications to come up [7]. Impressively inspired by the contemporary scientific breakthroughs in delineation of key biological mediators in fibrosis-associated diseases such as kinase signaling and myo-fibroblast activation [13,14], a particular spectrum of innovative medicine has emerged to be capable of combating fibrosis and cancer simultaneously [2,4,15]. In this light, the article herein presents an updated understanding of the recently approved drugs of a dual therapeutic efficacy on fibrosis and cancer, along with the underlying mechanisms of action (Table 1). 

## 2. Kinase Inhibitors

Protein kinases catalyze phosphorylation of tyrosine, serine and threonine residuals on membrane receptors or intra-cellular peptides to trigger adapter-selective protein binding and the downstream signaling cascades, thereby serving as a central mode in regulating a broad spectrum of physiological processes, covering growth, differentiation and metabolism [2,13]. Aberrant kinase activity is increasingly identified to contribute to the pathogenesis of various diseases including neoplastic and fibrotic disorders, among others. The outstanding therapeutic efficacy based upon selective kinase inhibition has highlighted targeted medicine as a contemporary milestone in clinical management and pharmaceutical innovation regarding oncology and beyond [4,16].

### 2.1. Platelet-Derived Growth Factor Receptor 

As the first success of targeted medicine, imatinib significantly advanced therapeutic strategies for chronic myeloid leukemia (CML) through inhibiting the breakpoint cluster region-Abelson (BCR-ABL) fusion protein, which drives constitutive tyrosine kinase activity. Impressively, imatinib has been able to extend five-year overall survival (OS) rate of CML patients beyond traditional treatments, thereby re-defining CML as a manageable disease [17]. On the other hand, imatinib is also a potent antagonist of c-kit receptor tyrosine (c-KIT) and PDGFRA kinases, both of which cause gastrointestinal stromal tumors (GIST) [18]. Going forward in this light, imatinib has been further revealed to exert a therapeutic efficacy for the patients with nephrogenic systemic fibrosis (NSF) as evidenced by reduced fibrosis which thus diminished skin thickness and improved joint function [19]. In addition to treating imatinib-resistant CML, the new generation BCR-ABL inhibitors developed later on, such as dasatinib and nilotinib, have also been approved to manage scleroderma and SSc [2,20]. In parallel, as a distinct inhibitor of PDGFR approved for managing a number of cancers, sunitinib has been shown to exert therapeutic efficacy on radiation-induced pulmonary fibrosis [21]. 

### 2.2. Platelet-Derived Growth Factor Receptor and Vascular Endothelial Growth Factor Receptor 

Nintedanib was initially discovered as an inhibitor of angiogenesis-associated kinases including PDGFRα, VEGFR and fibroblast growth factor receptor-1 (FGFR-1). Subsequently, this compound was noticed to down-regulate the accumulation of collagen while suppressing proliferation and differentiation of myofibroblasts [22]. Clinically, nintedanib has been demonstrated to exert a therapeutic efficacy on IPF patients as evidenced by a slower decline in pulmonary function and a lower exacerbating rate compared to those of the placebo control group. Moreover, it has been observed that adding nintedanib to traditional chemotherapy for the patients with non-small cell lung cancer (NSCLC) was able to improve the clinical outcomes in terms of longer OS and higher progression-free survival (PFS) [4,22].

In parallel, sorafenib represents another multi-kinase inhibitor against vascular endothelial growth factor receptor (VEGFR), PDGFβ and rapidly accelerated fibrosarcoma (Raf) kinase, thus diminishing angiogenesis and tumor growth. To date, this inhibitor has been utilized in the clinic to treat a variety of malignant diseases including lung cancer, renal carcinoma, and soft tissue sarcoma, being in particular the only non-surgical and non-radiological therapy that has delivered an impressive efficacy of significant improving survival to the patients with advanced HCC [23,24]. Besides, experimental studies also showed that sorafenib exerted anti-fibrotic effects on preclinical models of liver cirrhosis, consequently reducing intra-hepatic vascular resistance, portal hypertension, and ameliorating intra-hepatic fibrosis, inflammation as well as angiogenesis [25]. Clinically, it has been reported that sorafenib was able to decrease portal venous flow by up to 40% in cirrhotic patient with HCC, while having no side-effects of other anti-angiogenesis agents, such as bleeding [24]. 

### 2.3. Janus Kinase-Signal Transducers and Activators of Transcription 

Since being identified as a potent inhibitor of Janus kinase (JAK) phosphorylation in the biological modulating pathway of immune network and hematological cells, ruxolitinib was revealed to efficaciously down-regulate levels of a wide variety of fibrogenic and pro-inflammatory cytokines. In corollary, this JAK inhibitor has been approved for the treatment of patients with myelofibrosis based upon an impressive therapeutic benefit including significant improvement of total symptom score and two years’ survival rate in clinical studies [26,27]. Subsequently, the clinical applications of ruxolitinib were also expanded to deal with polycythemia vera, another myeloproliferative neoplasm [27]. Furthermore, it was revealed that JAK-signal transducers and activators of transcription 3 (STAT3) signaling pathways capable of augmenting proliferation and survival of cancer stem cells through induction of the transcriptional machinery, thus promoting resistance to anti-neoplastic medications [28]. In light of these discoveries, ruxolitinib has been added to chemotherapeutic agents for managing patients with metastatic pancreatic cancer unresponsive to classic drugs, and thus delivered an encouraging improvement of OS [29].

### 2.4. Mammalian Target of Rapamycin 

The phosphoinositide 3-kinase (PI3K)-AKT-*mammalian target of rapamycin* (mTOR) axis plays a significant role in orchestrating the homeostasis of a number of physiological activities including metabolism, differentiation and survival; this signaling pathway, with mTOR kinase as a master modulator, is frequently perturbated in various pathological conditions such as neoplastic and fibrotic diseases [50]. Representing the first mTOR inhibitor approved for clinical applications, rapamycin (sirolimus) was revealed to suppress activation of immune cells, myofibroblasts and TGF-β release, thus conferring the anti-inflammatory and anti-fibrotic effects on chronic kidney disease (CKD) and pulmonary fibrosis in animal models [2,50]. Interestingly, this inhibitor has further been verified as capable of preventing interstitial fibrosis through down-regulating angiogenesis and inflammation in the patients with renal transplantation [30]. On the other hand, in clinical settings regarding oncology, rapamycin was also observed to enhance remission induction of acute myeloid leukemia and to minimize skin cancer development in transplant recipients [21,32]. Moreover, everolimus, being identified as a mTOR complex1-selective inhibitor, was approved for the prevention of organ rejection following transplantation and treatment of several malignant indications including advanced kidney cancer [33]. Recently, it has been noticed that everolimus contributes to preserving the structure and function of transplanted kidneys through suppressing fibrotic processing in the clinic [35]. 

## 3. Cytokine Signaling Antagonists

There is consensus that fibrotic and neoplastic disorders are often linked to chronic inflammation in which cytokines function as a group of infamous mediators [6,51]. Activated fibroblasts/myofibroblasts are highly responsive to paracrine or autocrine-derived cytokines including PDGF, TGF-β, interleukine-6, tumor necrosis factor-α (TNF-α) and several chemokines [2,52]. TGF-β was revealed to induce the expression of α-smooth muscle cell actin (α-SMA) as a hallmark molecule of myofibroblasts, and to enhance collagen production as well as ECM accumulation [3]. In parallel, TNF-α could mediate adaptive immune responses and inflammation which might in turn promote fibrotic processing [52]. Besides, C-C cytokine receptor type 5 (CCR5) signaling axis was noticed to play a substantial role in driving the migration of hepatic stellate cells (HSC) in liver fibrosis [2]. 

### 3.1. Transforming Growth Factor-β 

A role of anti-pulmonary fibrosis by pirfenidone, a pyridine analogue, is traced back to two decades ago in experimental studies with animal models. It was later discovered that this compound is able to inhibit the nuclear translocation of intra-cellular proteins SMAD2/3, and thus to down-regulate TGF-β signaling activity, consequently suppressing many fibrotic phenotypes such as fibroblast proliferation, α-SMA expression and ECM accumulation [2,22]. Moreover, pirfenidone has recently been approved as an innovative therapy for IPF patients based on clinical trials demonstrating that this new drug conferred a therapeutic efficacy through reducing forced vital capacity (FVC) decline, delaying acute exacerbation and improving OS [22]. In addition, to circumvent the challenging co-morbidity issue of IPF with lung cancer, emerging evidence suggests that the anti-proliferative activity of pirfenidone may exert a synergistic effect with current chemotherapeutic regimens [5]. Of note, pirfenidone application appeared to significantly decrease the risk of lung cancer in patients with IPF according to retrospective data [35]. Impressively, peri-operative administration of pirfenidone has been revealed to be capable of preventing patients with concomitant IPF and lung cancer from life-threatening acute exacerbation after cancer surgery [36]. Moreover, while another TGF-β inhibitor LY2109761 exerted a strong anti-cancer effect, this compound has been repositioned to controlling radiation-induced pulmonary fibrosis through down-regulated inflammation and angiogenesis [53,54]. 

### 3.2. Tumor Necrosis Factor-α

Tumor necrosis factor-α inhibitors represent a contemporary targeted therapy for a spectrum of autoimmunity-mediated disorders such as rheumatoid arthritis and inflammatory bowel disease [51]. Beyond these medical fields, TNFα signaling pathway and the downstream biological network have also been shown to substantially contribute to cancer progression and fibroblast promotion [51,55,56]. In corollary, etanercept, a recombinant protein-based TNFα antagonist, was revealed to be well-tolerated and to decrease disease progression rates in patients with IPF [37]. On the other hand, at least for short-term application, this biological agent appeared to be able to synergistically enhance the therapeutic efficacy of existing medication in regard to controlling neoplastic progression [38]. Interestingly, as a small chemical compound capable of accelerating TNFα mRNA degradation and thus diminishing encoding of this cytokine protein, thalidomide was approved to treat autoimmunity-associated disease and malignant indications such as multiple myeloma; this compound has been recently shown to alleviate respiratory symptoms and to improve life quality in patients with IPF [39,40]. Moreover, while pomalidomide, a modified version of thalidomide with the same mechanism of action, was also approved for clinically managing multiple myeloma, this medication has shown therapeutic efficacy for myelofibrosis [39,41]. Additionally, thalidomide has also been revealed to improve clinical outcomes of patients with refractory inflammatory bowel disease (IBD) through diminishing TNFα and interleukine-12 expression [57]. Since inflammation is a shared mechanism by cancer and fibrosis, and in particular higher a morbidity of colon cancer is linked to IBD, the anti-inflammatory effects of thalidomide appear to at least in part contribute to its therapeutic roles against neoplastic and fibrotic disorders. 

### 3.3. C-C Cytokine Receptor Type 5

Belonging to the G-protein coupled receptor family, CCR5 represents a chemokine receptor predominately expressed on the surface of immune cells and certain malignant cells. Intriguingly, the human immunodeficiency virus-1 (HIV-1) frequently enters target cells upon binding its envelope glycoprotein gp120 to the CD4 receptor and CCR5 co-receptor [58]. Maraviroc, an imidazopyridine-derived small chemical compound, was discovered through high-throughput screening as a CCR5 antagonist to block the entry of HIV-1 into CD4 T-lymphocytes. Of note, maraviroc has been approved to treat HIV-1 infected humans based on the results from clinical trials showing that this compound significantly diminishes the viral copies and increased CD4 cell counting in blood of the patients [42,58]. Impressively, maraviroc was also demonstrated to be efficacious for controlling the progression of hepatic fibrosis in patients with HIV-1/hepatitis-C virus (HCV) co-infection [2,42]. In parallel, maraviroc-resulted CCR5 blocking was revealed to be capable of suppressing malignant cell metastasis of breast, gastric, and prostate in animal models [58]. Moreover, in the clinical setting, maraviroc has delivered objective therapeutic benefits including prolonged OS to the patients with advanced colorectal cancer [43]. 

## 4. Proteostasis Modulators

Functioning as essential biological machinery, proteostasis defines a dynamic processing covering protein expression, posttranslational modification, correct folding, interaction and degradation. It has been increasingly recognized that dysfunctional proteostasis often contributes to the common pathogenesis of various human diseases including cancer and fibrosis, among others [2,51,59]. Interestingly, the molecular components in certain phases of aberrant proteostasis, such as epigenetic regulation of peptide translation and proteasomal degradation of unwanted protein, have been validated as the therapeutic targets for developing innovative anti-tumor drugs successfully [51]. Moreover, emerging evidence demonstrates that these targeted agents can also be repositioned to confer clinical benefits to patients with fibrotic disorders [2,47].

### 4.1. Histone de-Acetylase Inhibitor

Given that acetylation of lysine amino acid in histone affects alterations of compact chromatin structure and thus gene expression, a high level of histone de-acetylation by HDAC over-expression induces translational repression and thereby a number of pathological conditions [2,51]. In this light, several HDAC inhibitors including vorinostat, romidepsin and chidamide have been developed and approved for T-cell lymphoma, followed by panobinostat for multiple myeloma [44,45]. The histone de-acetylase (HDAC)-targeted agents were revealed to result in neoplastic cell cycle arrest, cell death or differentiation, while tipping the tumor microenvironment through diminishing angiogenesis and modulating immune cells [45,51]. Clinically, these HDAC inhibitors have been demonstrated to deliver a therapeutic benefit by conferring an efficacious response rate of up to 30% in patients with indicated cancer types [44]. In parallel, the HDAC inhibitor romidepsin has been discovered to potently suppress fibroblast proliferation, myofibroblast differentiation, pulmonary fibrosis in animal models [45]. Moreover, another HDAC inhibitor panobinostat, at low doses over a long time, was also noted of being able to improve clinical outcomes of patients with primary myelofibrosis and post-polycythaemia vera/essential thrombocythaemia fibrosis [46]. 

### 4.2. Proteasomal Inhibitor

Representing the main housekeeping machinery for protein disposal and quality control, the ubiquitin-proteasome pathway is increasingly noted to substantially contribute to regulation of pathogenesis-linked mediators and thus biological phenotypes of relevant diseases [47,59]. In this light, the first proteasomal inhibitor bortezomib has been approved to treat patients with relapsed/refractory multiple myeloma due to the superior therapeutic efficacy over that of traditional medication in terms of improving PFS. Interestingly, bortezomib was also revealed to down-regulate TGF-β/Smad/collagen signaling and to induce myofibroblast/HSC apoptosis, consequently attenuating the severity of histological fibrosis of the lung, liver and kidney in animal models [47,48]. Moreover, to minimize the risk of lung infection upon corticosteroid treatment, bortezomib has instead been utilized to manage patients with concomitant cystofibrosis and multiple myeloma, impressively achieving a very good partial response without serious adverse events [49]. 

## 5. Perspective

The reciprocal association between fibrotic and neoplastic disorders has been noticed for decades through clinical observations and epidemiological investigations. While onco-pathogenesis frequently occurs following or with fibrosis exemplified by cirrhosis-linked hepatocellular carcinoma and cancer-associated fibroblasts in the tumor microenvironment, fibrotic disorders can conversely be induced by oncologic events such as skin sclerosis resulting from anti-cancer therapies of radiation and chemical agents [2,7,8,49]. Subsequently, accumulated pieces of biological evidence have revealed a number of aberrant molecular pathways that are shared by cancer and fibrosis, including oncogenic gene expressing signatures and epigenetic reprogramming among others [9,51]. In this light, novel targeted medicine has thus been developed to selectively modulate an array of functional proteins contributing to pathogenesis across these two medical conditions, thus conferring the optimal therapeutic benefits to patients with concomitant fibrotic and neoplastic disorders, in terms of lower costs and slighter toxicity than those of multi-drug combinations [2,51,60]. 

Whereas modulating the related kinase activities, cytokine signaling, and protein homeostasis has come up with a productive portfolio of efficacious medications to treat fibrosis and cancer simultaneously, such interdisciplinary scientific interests across these two disease fields are currently going beyond the established therapeutic targets, inspiring innovative preclinical studies [2,51]. In this sense, while aberrant activation of the developmental biological pathway Wnt/β-catenin was recognized to facilitate cancer stem cells and pulmonary fibrosis [61,62], TNF related apoptosis-inducing ligand (TRAIL) signaling cascade is emerging as a key mediator for malignant cell death as well as myofibroblast inhibition [63]. Meanwhile, the lysophosphatidic acid (LPA) pathway is emerging as an important mediator in the pathogenesis of cancer and fibrosis; of note, the inhibitors of LPA-producing enzyme autotaxin and LPA receptors have showed an encouraging efficacy during phase II clinical trials in patients with pulmonary fibrosis [64,65]. In addition, several microRNAs (miRs) were revealed to play a comprehensive promoting role in co-pathogenesis of fibrotic and neoplastic disorders; moreover, specific oligo-nucleotides of anti-miR-21 have been in preclinical investigations for dual targeting across the two medical conditions recently [2,66]. Hence, despite it having been contemporarily proposed to treat a severe disease by means of multi-targeted approaches such as combined therapies, insights derived herein also highlight an exceptional perspective that novel medicine with a dual efficacy can be designed and developed through modulating a shared patho-biological pathway by distinct medical conditions such as fibrosis and cancer.

## Figures and Tables

**Table 1:** Representative medicine of dual targeting fibrosis and cancer.

Group	Target	Drug Name	Fibrotic Indication	Neoplastic Indication	References
Kinase inhibitor	PDGFR	Imatinib	Nephrogenic systemic fibrosis	CML, gastrointestinal	
		Nilotinib	SSc	Stromal tumor	[2,16,17,18,19,20]
		Dasatinib	Sclerodoma		
	PDGFR & VEGFR	Nintedanib	IPF	NSCLC	[4,22]
		Sorafenib	Liver cirhosis	HCC	[23,24,25]
	JAK-STAT	Ruxolitinib	Myelofibrosis	Polycythemia vera, pancreatic cancer	[26,27,28,29]
	mTOR	Sirolimus	Renal fibrosis	renal cancer	[30,31,32,33,34]
		Everolimus			
Cytokine signaling antagonists	TGF-β	Pirfenidone	IPF	Lung cancer	[22,35,36]
	TNF-α	Etanercept	IPF	Lymphoma	[37,38]
		Thalidomide	IPF	Multiple myeloma	[39,40,41]
		Pomalidomide	Myelofibrosis	Multiple myeloma	
	CCR5	Maraviroc	Hepatic fibrosis	Colorectal cancer	[42,43]
Proteostasis modulator	HDAC	Romidepsin	Pulmonary fibrosis	T-cell lymphoma	[44,45,46]
		Panobinostat	Myelofibrosis	Multiple myeloma	
	Proteasome	Bortezomib	Cystofibrosis	Multiple myeloma	[47,48,49]

PDGFR: platelet-derived growth factor receptor, SSc: systemic sclerosis, CML: chronic myeloid leukemia, VEGFR: vascular endothelial growth factor receptor, IPF: idiopathic pulmonary fibrosis, NSCLC: non-small cell lung carcinoma, HCC: hepato-cellular carcinoma, JAK-STAT: Janus kinase-signal transducers and activators of transcription, mTOR: mammalian target of rapamycin, TGF-β: transforming growth factor-β, TNF-α: tumor necrosis factor-α, CCR5: C-C cytokine receptor type 5, HDAC: histone de-acetylase.
